# Assessing the sustainability of Rwanda’s mass drug administration program for schistosomiasis and soil-transmitted helminthiasis: A mixed-methods application of the program sustainability assessment tool

**DOI:** 10.1371/journal.pntd.0014455

**Published:** 2026-06-25

**Authors:** Christian Mazimpaka, Davidson H. Hamer, Eugene Ruberanziza, Kevin James Lane, Ladislas Nshimiyimana, Jean Bosco Mbonigaba, Aimable Mbituyumuremyi, Donald M. Thea, Veronika J. Wirtz

**Affiliations:** 1 Department of Global Health, Boston University School of Public Health, Boston, Massachusetts, United States of America; 2 Ministry of Health, Kigali, Rwanda; 3 Section of Infectious Diseases, Department of Medicine, Boston University Chobanian & Avedisian School of Medicine, Boston, Massachusetts, United States of America; 4 END Fund, New York, New York, United States of America; 5 Department of Environmental Health, Boston University School of Public Health, Boston, Massachusetts, United States of America; 6 Division of Malaria and Other Parasitic Diseases, Rwanda Biomedical Centre, Ministry of Health, Kigali, Rwanda; George Washington University School of Medicine and Health Sciences, UNITED STATES OF AMERICA

## Abstract

**Background:**

Mass drug administration (MDA) is a key strategy for controlling schistosomiasis and soil-transmitted helminthiasis in low-resource settings. Assessing its long-term sustainability is important for maintaining and expanding disease control achievements. This study examined the sustainability capacity of Rwanda’s MDA program using the Program Sustainability Assessment Tool (PSAT).

**Methodology:**

A mixed-methods approach was used. Twenty-one key informants, including government officials, donors, and local stakeholders, participated in qualitative interviews, and 16 of them also completed the PSAT quantitative survey. Qualitative data were thematically analyzed, while quantitative findings from the survey were used to calculate domain-specific and overall sustainability scores.

**Findings:**

The overall Rwanda MDA program sustainability score was 3.75 out of 5. Partnerships scored the highest at 4.1, with qualitative data emphasizing strong collaboration among government agencies, international organizations, and community stakeholders. Political support and program evaluation both received a score of 3.9, with qualitative data emphasizing good policy alignment, but some issues with data accuracy. Funding stability was the weakest domain, scoring 3.3, and qualitative data highlighted reliance on external donors. Strategic planning scored 3.5, with qualitative data highlighting limited development of long-term financial strategies and the absence of a dedicated sustainability plan.

**Conclusion:**

This assessment revealed a moderate sustainability capacity for the MDA program, characterized by strong political support, active partnerships, and promising adaptations, including the expansion of treatment coverage to adults. However, dependence on donor funding, staffing shortages, and data management issues continue to pose a risk to the program’s sustainability. Improving domestic resource allocation, investing in human resources, and strengthening data systems could enhance the program’s capacity for sustainability.

## Author summary

This study evaluated the sustainability of Rwanda’s mass drug administration (MDA) program for schistosomiasis and soil-transmitted helminthiasis. Using interviews and a standardized sustainability tool (PSAT), we assessed the program’s current sustainability capacity. The results show that the program benefits from strong political support and strong partnerships among government, donors, and communities. However, funding stability and long-term planning are weak due to heavy reliance on donors and limited domestic financing. The overall program sustainability score was 3.75 out of 5, indicating moderate sustainability. To sustain progress, Rwanda must improve domestic resource allocation to the program, invest in workforce capacity, and strengthen data systems to ensure the continued delivery and expansion of MDA.

## Introduction

Neglected tropical diseases (NTDs) remain a significant global health challenge, disproportionately affecting impoverished communities [1]. Schistosomiasis (SCH) and soil-transmitted helminthiasis (STH) are among the most prevalent NTDs, affecting millions of people worldwide [2,3]. Both diseases are strongly associated with inadequate sanitation and limited access to clean water, resulting in chronic infections that can lead to anemia, malnutrition, and impaired physical and cognitive development [2,3].

In Rwanda, SCH and STH are more prevalent types of NTDs [4]. National mapping conducted in 2007–2008 estimated a 65.8% prevalence of STH nationwide, which declined to 45% in a 2014 follow-up among school-aged children (5–15 years) [4,5]. SCH mapping during 2007–2008 showed a national prevalence of 2.7%. However, the use of more sensitive diagnostic testing in 2014 revealed a broader geographic distribution, with a national prevalence of 7.4% when Point-of-Care Circulating Cathodic Antigen (POC CCA) trace results were counted negative [6]. Most recent published national prevalence data from 2020 show that SCH prevalence among school-age children has declined to 1.7% using the Kato-Katz method and 9.1% when the POC CCA traces are counted as negative, while the STH prevalence was 38.7% [7]. The government of Rwanda, through the national NTD program in collaboration with local and international organizations, has been conducting a series of mass drug administration (MDA) efforts since 2008 to reduce the prevalence of both diseases.

MDA involves regularly distributing antiparasitic medications, such as praziquantel for SCH and albendazole or mebendazole for STH, to entire at-risk populations, including schoolchildren and adults in high-prevalence districts, regardless of infection status [8,9]. In Rwanda, community health workers, teachers, and healthcare providers are responsible for distributing drugs [4]. STH MDA is delivered twice each year to those ≥1 years (including adults), and SCH MDA once yearly to those ≥5 years (including adults); both are integrated into the biannual Maternal and Child Health (MCH) Week; a week-long national campaign that provides MCH services such as immunization, vitamin A, growth monitoring, nutrition counseling, antenatal and postnatal check-ins, iron–folate for pregnant women, and family planning counseling among others [10].

Since its initiation in 2008, the MDA program has evolved from a campaign-based intervention into a nationally coordinated platform embedded within existing service delivery structures [4]. Its implementation has depended on multiple operational components beyond drug distribution, including planning, supervision, community mobilization, training, and monitoring at the community, school, and health facility levels. However, domestic program funding remains a challenge. Data from the Rwanda Biomedical Center shows domestic allocations for the NTDs program increased between 2019/2020 and 2022/2023, but external funding continued to dominate, accounting for 97.2% of reported financing in 2022/2023 and 97.7% over the four-year period.

As the program continues to increase its reach, the main challenge is whether it can be sustained over time. Program sustainability depends not only on the availability of MDA medicine but also on other factors such as financing, government ownership, adaptation, community engagement, data use, strategic planning, and organizational capacity. Evaluating these dimensions is important for identifying barriers to sustainability and for informing strategies to strengthen the program’s long-term contribution to NTD control and elimination. The Program Sustainability Assessment Tool (PSAT) offers a structured approach to assessing sustainability capacity across these key areas [11].

This study applied a mixed-methods approach, guided by the PSAT, to assess the capacity for sustainability of Rwanda’s MDA program for SCH and STH. It examined how political support, partnerships, funding, evaluation, communication, adaptation, and strategic planning shape the program’s ability to maintain and expand progress. While identifying core strengths and vulnerabilities, the study aimed to inform policymakers and stakeholders on strategies to solidify MDA’s role in reducing NTD burdens and creating a more resilient, self-sustaining public health MDA program.

## Methods

**Ethical statement:** Ethical approval was obtained from the Rwanda National Ethics Committee (RNEC 471/2024) and the Boston University Medical Campus Institutional Review Board (H-45007). All participants were adults, participation was voluntary, and written informed consent was obtained prior to data collection. Interviews were audio-recorded with consent, de-identified for analysis, and stored on a password-protected computer.

**Study design:** A mixed-methods approach was utilized to evaluate the sustainability capacity of Rwanda’s MDA program. This design combined both quantitative and qualitative data collected using a modified PSAT framework.

**Study population and sampling:** The study population comprised 21 stakeholders in the health system directly involved in Rwanda’s NTD program, including government officials/public-sector actors (58.8%), donor or multilateral representatives (11.8%), program managers or technical leads (52.9%), and implementers involved in service delivery or field coordination (47.1%). Selection was based on their decision-making roles and expertise in MDA activities. Snowball sampling was also used, in which participants interviewed recommended additional key informants with knowledge of the program.

**Data collection tool and adaptation:** The Program Sustainability Assessment Tool (PSAT), developed by the Center for Public Health Systems Science at Washington University in St. Louis, assesses a program’s capacity for sustainability across eight domains: political support, funding stability, partnerships, organizational capacity, program evaluation, program adaptation, communications, and strategic planning [11] The original tool consists of standardized Likert-type items organized into five items per domain. For this study, we used the low- and middle-income country-adapted version developed by Gizaw et al., which retained the original eight-domain structure while modifying wording for resource-limited settings and using a 5-point Likert response scale ranging from 1 (strongly disagree) to 5 (strongly agree) [12].

Building on this adapted version, we made only minor contextual refinements to improve applicability to Rwanda’s MDA program for schistosomiasis and soil-transmitted helminthiasis. These refinements were limited to wording changes that reflected the local MDA context, health system structure, and relevant stakeholder groups, without altering the number of items, the response scale, or the tool’s underlying constructs. In addition, we added domain-specific open-ended questions, informed by the mixed-methods application of the PSAT described by Stoll et al., to allow participants to explain and contextualize their ratings [13]. A marked version of the adapted PSAT survey, with minor contextual wording changes highlighted in red, is provided in S1 Appendix (Supporting Information). The accompanying open-ended questions are provided in S2 Appendix (Supporting Information).

**Collection procedure:** Two weeks prior to data collection, participants were contacted via email to schedule their survey completion and interview. The PSAT survey required to be completed electronically (via Qualtrics) at least 24 hours before the interview. If the survey was not completed in advance, participants filled it out prior to the interview. All interviews were conducted in person, recorded, and transcribed verbatim in Kinyarwanda; the transcripts were then translated into English.

**Data analysis:** Quantitative data were aggregated, and mean scores with standard deviations (SD) were calculated for each PSAT domain. For this study, we interpret mean scores as follows: ≥ 4.0 as strong or acceptable, 3.0-3.9 as moderate, and <3.0 as weak. Qualitative data were analyzed using a deductive coding approach aligned with PSAT domains.

Qualitative data were analyzed deductively using the eight PSAT domains as an a priori coding framework. Transcripts were reviewed systematically, relevant excerpts were coded to the corresponding domain(s), and coded data were organized in Excel matrices to identify recurring themes within and across participants. Coding discrepancies were resolved through iterative review. To integrate the mixed-methods findings, a domain-by-domain joint display was developed in which each PSAT domain was examined by comparing quantitative scores with corresponding qualitative themes and illustrative quotes. This process allowed the qualitative findings to explain, contextualize, and elaborate the survey results, and to identify areas of convergence and discrepancy across methods.

## Results

**Fig 1:**
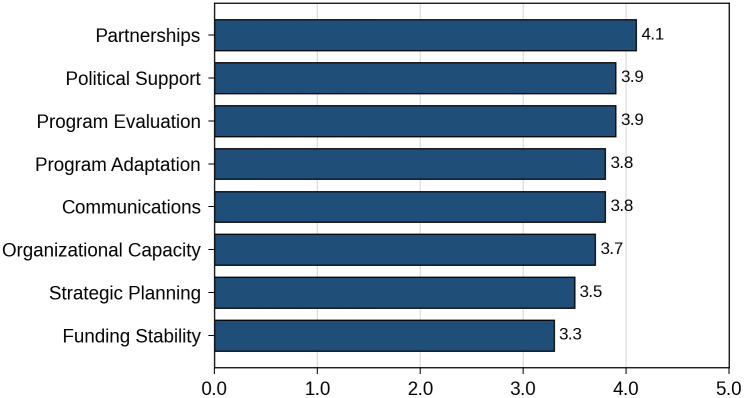
Program sustainability capacity across key domains.

A total of 21 key informants participated in qualitative interviews, with 17 also completing the PSAT survey. The Rwanda MDA program’s overall sustainability score was 3.75 out of 5. The highest-rated domain was partnerships (average = 4.1), followed by political support and program evaluation (with an average score of 3.9). The lowest-rated domains were funding stability (average = 3.3) and strategic planning (average = 3.5) (Fig 1, Table 1).

**Table 1:** Domain-specific scores for program capacity and sustainability.

Domain		Score; SD
Partnerships (Domain Score Average = 4.1, Domain Average SD: 0.92)	Diverse community organizations (government, private, non-profit and community organizations) are invested in the success of the program.	4.12; 0.70
	The program communicates with community leaders. from diverse organizations (government, private, non-profit and community organizations)	4.41; 0.80
	Diverse organizations (government, private, non-profit and community organizations) are engaged in the development of program goals	3.94; 1.03
	Local leaders from diverse organizations (government, private, nonprofit and community organizations) are engaged in the development of program activities	3.88; 1.11
	Diverse organizations (government, private, non-profit and community organizations) are engaged in implementation of program activities	4.12; 0.99
Political support (Domain Average Score = 3.9, Domain Average SD: 1.01)	Political champions advocate for the program	3.88; 1.31
	The program has strong champions with the ability to garner resources	3.40; 0.99
	The program has political support within the larger organization	4.19; 0.83
	The program has political support from outside of the organization	4.13; 0.96
	The program has strong advocacy support	3.80; 0.94
Program Evaluation (Domain Average = 3.9, Domain Average SD: 0.96)	The program has the capacity for quality program evaluation	3.47; 0.87
	The program reports short term, intermediate and long-term outcomes	3.88; 0.99
	Evaluation results inform program planning and implementation	3.94; 0.90
	Program evaluation results are used to demonstrate successes to funders and other key stakeholders	4.12; 1.05
	The program provides strong evidence to the public that the program works	3.94; 0.97
Program Adaptation (Domain Average = 3.8, Domain Average SD: 0.76)	The program periodically reviews the evidence base	3.29; 0.77
	The program adapts strategies as needed	3.94; 0.56
	The program adapts to new science	3.88; 0.86
	The program proactively adapts to changes in the environment	3.94; 0.90
	The program makes decisions about which components are ineffective and should not continue.	3.82; 0.73
Communications (Domain Average = 3.8. Domain Average SD: 0.95)	The program has communication strategies to secure and maintain public support from local stakeholders (government, private, non-profit and community organizations)	3.71; 1.10
	Program staff communicate the need for the program to the public	3.94; 0.90
	The program is marketed in a way that generates interest	3.24; 1.15
	The program increases community awareness of the issue	4.12; 0.78
	The program demonstrated its value to local stakeholders (government, private, non-profit and community organizations)	4.24; 0.83
Organizational Capacity (Domain Average Score = 3.7, Domain Average SD: 1.03)	The program is well integrated into the operations of the organization	3.82; 0.88
	Organizational systems are in place to support the various program needs	3.82; 0.88
	Leadership effectively articulates the vision of the program to external partners	4.06; 0.97
	Leadership efficiently manages staff and other resources	3.65; 1.22
	The program has adequate staff to complete the program’s goals	3.06; 1.20
Strategic Planning (Domain Average = 3.5, Domain Average SD: 0.94)	The program plans for future resource needs	3.76; 1.03
	The program has a long-term financial plan	3.24; 0.90
	The program has a sustainability plan	2.82; 1.01
	The program’s goals are understood by all stakeholders (government, private, non-profit and community organizations)	3.88; 0.93
	The program clearly outlines roles and responsibilities for all stakeholders. (Government, private, non-profit and community organizations)	3.82; 0.81
Funding stability (Domain Average Score = 3.3, Domain Average SD: 0.99)	The program exists in a supportive state economic setting	3.76; 0.83
	The program implements policies to help ensure sustained funding beyond initial funding cycle	3.35; 1.06
	The program is funded through balanced contribution from variety of sources.	3.35; 1.00
	The program has a combination of stable beyond initial funding cycle	3.06; 1.09
	The program has flexible, unrestricted funding beyond initial funding cycle	3.00; 1.00

SD = standard deviation

### Partnerships

**Fig 2:**
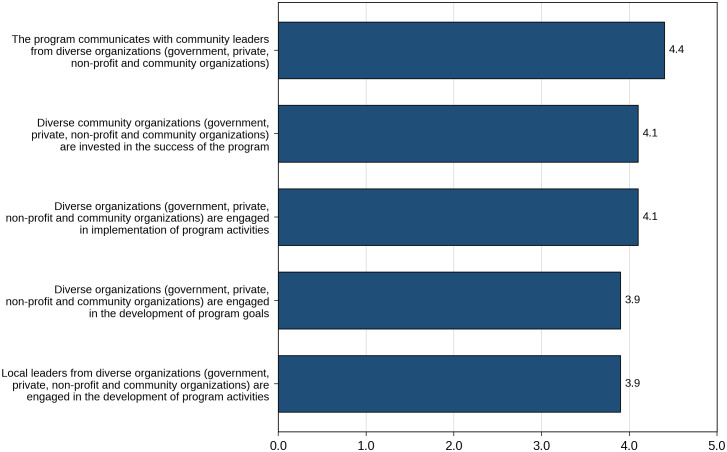
Program Sustainability Capacity Evaluation: Partnerships.

Partnership had a domain average score of 4.1 and a domain average SD of 0.92. Results indicate that diverse organizations are invested in the program’s success (average 4.1, SD = 0.697), and the program communicates effectively with community leaders from various sectors (average 4.4, SD = 0.795) (Fig 2) (Table 1). From qualitative findings, participants described the MDA program as being supported by a broad network of government, partner, donor, and community actors with complementary roles in policy guidance, implementation support, commodity provision, and community mobilization. Across interviews, respondents consistently emphasized the importance of local leadership and community-level actors in building trust and encouraging participation during campaigns. As one participant explained, *“Local leaders are the most important because they are the ones in direct contact with the population. They are the ones in charge of mobilizing”* (Executive Director-Local NGO).

### Political support

**Fig 3:**
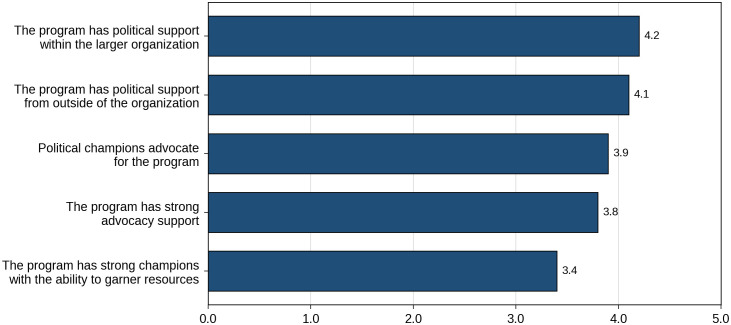
Program Sustainability Capacity Evaluation: Political Support.

The quantitative results indicate strong overall political support for the MDA program (domain average score = 3.9, domain average SD: 1.01), particularly for political support within the organization (average = 4.2, SD = 0.83) and from external entities (average = 4.1, SD = 0.96) (Fig 3) (Table 1).

Qualitative findings generally supported these results while also identifying an important nuance. Participants described strong political support for the MDA program at the national and district levels, largely because it aligns with broader government priorities, including child health and stunting reduction, and is tracked through locally performance contracts, known as imihigo. At the same time, several respondents suggested that this performance pressure could affect target-setting and reporting practices, particularly when districts sought to ensure targets were achieved. As one participant stated, “*Politically, this program is very sensitive... the government puts it in performance contracts (imihigo)... to reduce mother and child mortality and stunting prevalence.”* (Executive Director-Local NGO). *Another noted, “Sometimes, they put low targets because they want to easily hit targets, set in performance contracts (imihigo)*” (Data Analyst-Local NGO).

### Program evaluation

**Fig 4:**
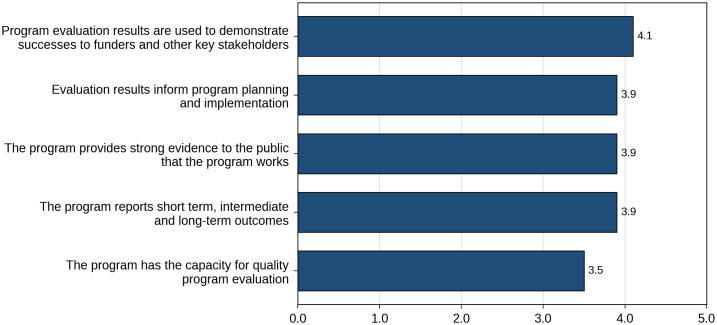
Program Sustainability Capacity Evaluation-Program Evaluation.

Program evaluation was assessed with a domain average score of 3.9 and a domain average SD of 0.96. The highest score was for using evaluation results to demonstrate successes to funders and key stakeholders (average = 4.1, SD = 1.1). The lowest score was for the program’s capacity for quality program evaluation (average = 3.5, SD = 0.9) (Fig 4) (Table 1).

Qualitative findings supported these results and highlighted several operational weaknesses in program evaluation. Participants described a centralized data management process that limited timely feedback to districts and health facilities, which in turn **affects** local use of data for decision-making. Respondents also identified persistent data quality challenges, especially in estimating the denominator and target populations for MDA, with districts using different approaches and limited standardization across levels. As one participant noted, *“RBC (Rwanda Biomedical Centre) collects the data from the Maternal and Child Health (MCH) week; they analyze them, make a report, and share it with the districts... If it were decentralized, the district would own it”* (Health Technical Advisor-NGO). Another explained, *“To estimate MDA targets, some use assessments by community health workers, others use ‘ubudehe’ (the government’s socio-economic categorization system) categorization lists from the Ministry of Local Government (MINALOC), and others use lists from the National Institute of Statistics of Rwanda (NISR)”* (Data analyst-NGO).

### Program adaptation

**Fig 5:**
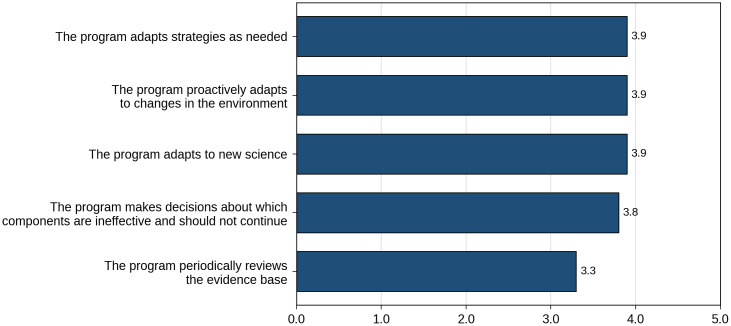
Program Sustainability Capacity Evaluation-Political Adaptation.

Program adaptation scored a domain average score of 3.8 and a domain average SD of 0.76. High scores were observed in adapting strategies as needed, adapting to new science, and proactively responding to environmental changes (each averaging 3.9). A lower score was noted for periodically reviewing the evidence base (average = 3.3) (Fig 5) (Table 1).

Qualitative findings highlighted important adaptations to the program over the years. Notably, the MDA program expanded its treatment regimen to include adults due to the high prevalence, aiming to accelerate disease reduction across all age groups and reduce transmission from this group to children. Additionally, integrating MDA indicators into the Health Management Information System (HMIS) transitioned data collection from paper-based to digital platforms. Participants also highlighted that the program decentralized tablet distribution from the sector to village-level sites, increasing accessibility, particularly in rural areas, by reducing recipients’ travel distances. *“In terms of adaptation, what was innovative is the inclusion of adults... we had to adapt and include them in the treatment for us to accelerate the reduction of the prevalence.”* (Government, Senior Officer #1).

### Communications

**Fig 6:**
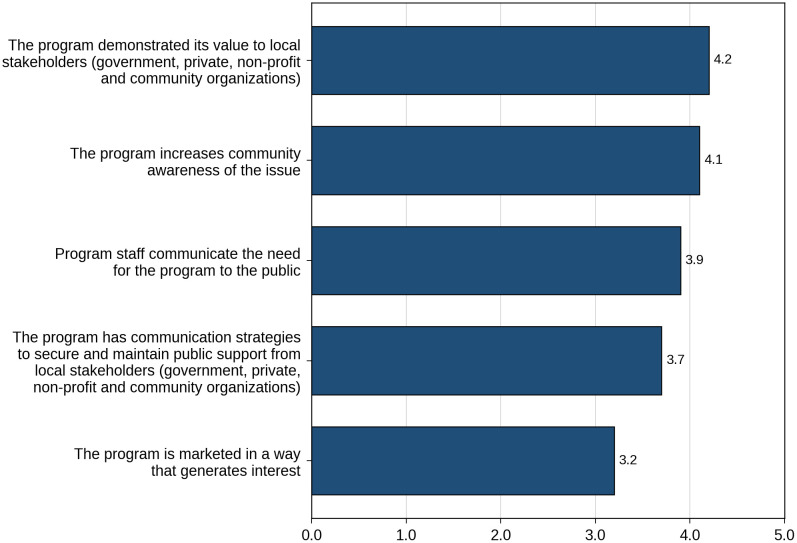
Program Sustainability Capacity Evaluation-Communications.

Communications were assessed with a domain average score of 3.8 and a domain average SD of 0.95. High scores were noted for the program’s ability to demonstrate its value to local stakeholders (average = 4.2, SD = 0.8) and increase community awareness of the issue (average = 4.1, SD = 0.8). A lower domain score was found for marketing the program in a way that generates interest (average = 3.2, SD = 1.1) (Fig 6) (Table 1).

The qualitative findings highlighted various communication tools and strategies, including traditional media (radio, TV) and social media, as well as collaboration with journalists and influencers trained on NTDs to disseminate information widely. Community-based channels are also utilized, with local authorities and community health workers conducting house-to-house sensitization and using megaphones at gatherings. Visibility materials such as flyers, t-shirts, and performances by the “Urunana” soap opera troupe enhance public engagement. Participants emphasized the role of communication in increasing medicine uptake and promoting behavior change. However, they noted gaps in maintaining consistent communication beyond campaign periods. One participant stated, “*In between MDA campaigns, people return to their old ways, forgetting all the messages we gave them. It would be better to remind them constantly so that they adopt preventive behaviors*. " (District Officer, Rwanda NGO Forum)

### Organizational capacity

**Fig 7:**
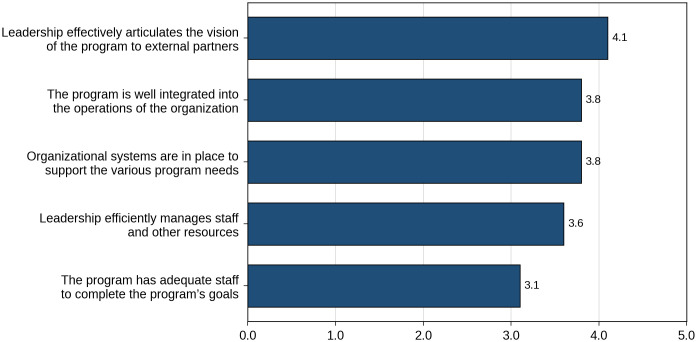
Program Sustainability Capacity Evaluation-Organizational Capacity.

The MDA program’s organizational capacity was assessed with a domain average score of 3.7 and a domain average SD of 1.03. The highest score was for leadership effectively articulating the program’s vision to external partners (average = 4.1, SD = 1.0). The lowest score was for the program’s adequate staff (average = 3.1, SD = 1.2) (Fig 7) (Table 1).

Qualitative findings supported these results, particularly regarding staffing constraints. Participants consistently described inadequate human resources as a key limitation, especially for follow-up, supervision, advocacy, and coordination when multiple activities occurred at the same time. Respondents noted that these staffing gaps were most apparent at central level, where limited personnel reduced the program’s ability to mobilize resources and sustain high-level engagement despite strong leadership and government support. As one participant stated, *“Understaffing means they are unable to raise their voice and advocate at the high level for things to be sped up”* (Program Director-Donor). Another noted, *“The government support is there... but the challenge comes when the program needs more human resources for follow-up and supervision, especially when there are overlapping activities”* (Government staff, Senior Officer #2).

### Strategic planning

**Fig 8:**
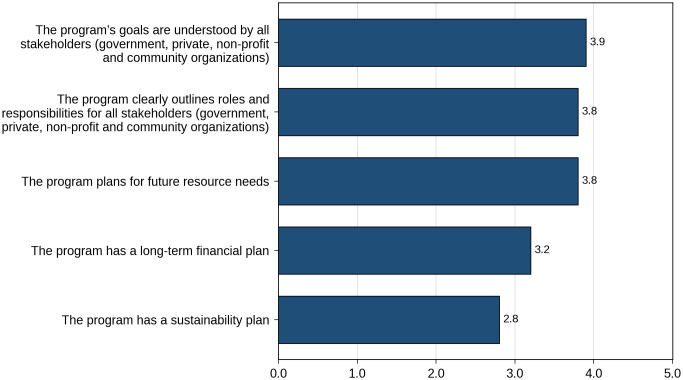
Program Sustainability Capacity Evaluation-Strategic Planning.

Strategic planning was assessed with a domain average score of 3.5 and a domain average SD of 0.94. The highest scores were for the program’s goals being understood by all stakeholders (average = 3.9, SD = 0.9). The lower scores were about long-term financial plan (average = 3.2, SD = 0.9) and possessing a sustainability plan for the MDA program (average = 2.8, SD = 1.0) (Fig 8) (Table 1).

Qualitative findings corroborated these results and pointed to an important gap in formal sustainability planning. Participants generally described the program as operating within broader strategic planning frameworks, but many indicated that sustainability planning was a small section embedded in those plans rather than formalized as a clear, standalone component. As one participant stated, *“The current strategic plan does not include a sustainability plan, and we never had a standalone sustainability plan for the program, but one is being incorporated into the new strategic plan under development.”* (Government staff, Senior Officer #1).

### Funding stability

**Fig 9:**
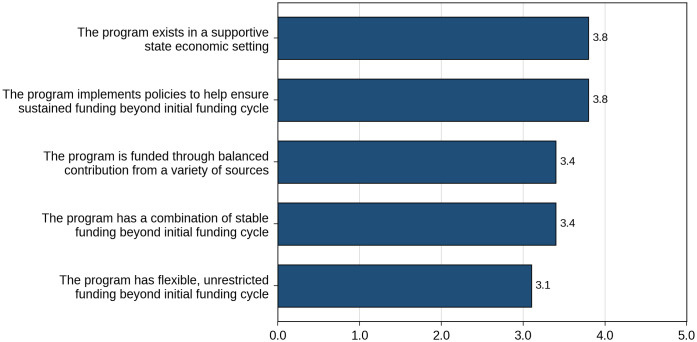
Program Sustainability Capacity Evaluation-Funding Stability.

Funding stability was the weakest and lowest-scoring domain in our assessment of the program, with an average score of 3.3 and a domain average SD of 0.99. The gaps were mostly observed for questions related to the program having stable (average 3.1, SD = 1.0) and flexible (average 3.0, SD = 1.0) funding (Fig 9) (Table 1). Qualitative findings aligned with these results, showing that the program relies heavily on external donors, such as the WHO and the END Fund, for acquiring drugs, while domestic funding mostly covers MDA operational costs. The government also contributes to the procurement of adult treatments and operational costs, but the contributions are not yet sufficient to fully sustain the program. As one participant noted, *“Most of the time, government budget allocation on NTDs is low compared with others such as malaria, HIV, Tuberculosis... but we are encouraging them [the government] to continue increasing their contribution every fiscal year”* (Program Director, Donor).

## Discussion

As our findings highlight, high-level political support has influenced the integration of Rwanda MDA into national health priorities and the district performance contracts. Such support aligns with global recommendations for securing leadership buy-in to sustain public health interventions [14,15]. This high political support, while important for the MDA program, also appeared to create unintended consequences. Specifically, some interviewees perceived that performance-based contracts (imihigo) may, at times, inadvertently incentivize modest district targets to ensure alignment with national MDA indicators. Similar practices have been documented in other global health contexts, where strong political support incentives can inadvertently distort local-level data reporting practices to meet predetermined benchmarks rather than accurately reflect on-the-ground achievements [16]. Such pressures can undermine data quality, skew resource allocation, and potentially erode trust among stakeholders.

Furthermore, the program’s reliance on external donor funding creates vulnerabilities that extend beyond financial instability. Heavy dependence on external sources often constrains strategic investments in human resources, limiting the program’s ability to recruit, train, and retain skilled personnel [17,18]. As a result, shortages in qualified staff may reduce oversight, affect continuous quality improvement, and limit routine data collection and reporting efforts. Similar patterns are observed in other low-resource settings where donor-driven priorities shape resource allocations, sometimes leaving insufficient flexibility for strengthening local management capacities and data system [19]. Over time, such gaps can compromise data quality, affect evidence-based decision-making, and risk misalignment of program strategies. Building balanced domestic funding streams, supported by strategic health financing policies, can enhance long-term resiliency, improve workforce stability, and ensure higher-quality data to guide sustainable scale-up of NTD control efforts [17–19].

Beyond immediate resource constraints, prolonged donor reliance affects the integration of the MDA program in the national health system and its long-term strategic planning. Without guaranteed financing, program leadership struggles to establish stable workforce pipelines, resulting in high staff turnover and interruptions to skill-building efforts. This, in turn, erodes organizational memory, limits institutional learning, and reduces responsiveness to evolving program needs. Similarly, while evaluation activities may be prioritized in principle, persistent funding instability affects the development of locally owned evaluation frameworks and weakens the integration of data-driven insights into routine decision-making. Such conditions risk cultivating a cycle where evidence generation and application remain donor-dependent [20]. Investing in diversified funding streams, whether through enhanced domestic financing or innovative public-private partnerships, may gradually alleviate these structural constraints [21].

While the Rwanda MDA program successfully adapted key strategies such as expanding treatment coverage to include adults and integrating NTD data into the national HMIS, sustaining these initiatives remains challenging without stable, diversified funding. Although MDA drugs are largely donated, dependence on external support for medicine supply can still disrupt program continuity when delivery timelines and quantities are uncertain; at the same time, limited domestic financing constrains the program’s ability to improve data quality, strengthen data use, and respond to changing operational needs [17,18]. Moreover, although results showed that campaign-based communication efforts have enhanced community awareness, episodic outreach alone fails to enhance routine health-seeking behaviors. Sustainable change requires integrating ongoing, evidence-based messaging into routine health services and broader Social Behavior Change Communication (SBCC) frameworks [22]. Aligning these approaches with national health education strategies can help establish continuous dialogue, reinforce prevention norms, and reduce reliance on short-lived, donor-dependent interventions.

Importantly, Rwanda has recently codified decentralization and integration of NTD services, including MDA through a national guideline in 2020 that define clear roles from district to village and schools, embed delivery in routine platforms (community assemblies, school sessions, monthly CHW meetings), route commodities/data through eLMIS/HMIS, and explicitly leverage supervision fees from other health programs to fund integrated supervision [23]. However, implementation has not been adopted because donor-funded MCH Week continued to host MDA. With the recent USAID funding cut, RBC, through UNICEF, is transitioning all MCH Week services into routine care, and MDA is also expected to be integrated into routine care. This transition may provide a near-term opportunity to strengthen country ownership and sustainability by integrating MDA into routine budgets, supervision, and data-use cycles.

This study has some limitations. Firstly, relying on self-reported data, especially from policymakers and government employees directly involved in program implementation, raises the risk of social desirability bias. However, this mixed-methods approach to the PSAT provides a more thorough understanding of sustainability capacity than a single method alone. Additionally, including informants from both government agencies and NGOs helped balance the perspectives on the program. Secondly, although the PSAT is an established tool, the version used in this study was slightly adapted for the Rwanda SCH/STH MDA context and was not formally validated in this setting. The quantitative findings should therefore be interpreted as context-specific indicators of sustainability capacity and considered alongside the qualitative results.

This mixed-methods assessment identified a moderate level of sustainability capacity in Rwanda’s MDA program for SCH and STH. Strong political support, active partnerships, and effective communication highlight the program’s potential to sustain progress. However, reliance on donor funding, staffing shortages, and data management issues expose vulnerabilities to long-term sustainability. By addressing these gaps, the MDA program can evolve into a fully integrated, system-embedded intervention. To our knowledge, this is the first study to use the PSAT in Rwanda to evaluate the sustainability of a health program. By providing initial evidence and identifying programmatic weaknesses, these findings can help guide strategic improvements and inspire policymakers to include systematic sustainability assessments in other Rwandan health initiatives.

## Supporting information

S1 Appendix Modified Program Sustainability Assessment Tool (PSAT): Capacity for Sustainability Assessment. (DOCX)

S2 Appendix Interview Guide for Capacity for Sustainability Assessment. (DOCX)

S3 Appendix Modified Program Sustainability Assessment Tool (PSAT) Survey Data. (XLSX)
