# Supplementary material for: Assessing the sustainability of Rwanda’s mass drug administration program for schistosomiasis and soil-transmitted helminthiasis: A mixed-methods application of the program sustainability assessment tool
Source: PLoS Negl Trop Dis. 2026 Jun 25;20(6):e0014455. doi: 10.1371/journal.pntd.0014455 (PMC13298938; doi:10.1371/journal.pntd.0014455)
Supplement: S1 Appendix — (DOCX) [file pntd.0014455.s001.docx]

S1. Appendix. Modified Program Sustainability Assessment Tool (PSAT): Capacity for Sustainability Assessment.

| **Political Support: Internal and external political environments that support your program** | **Strongly disagree** | **disagree** | **Neither agree nor disagree** | **agree** | **Strongly agree** |
| --- | --- | --- | --- | --- | --- |
| 1. Political champions advocate for the program. | 1 | 2 | 3 | 4 | 5 |
| 2. The program has strong champions with the ability to garner resources. | 1 | 2 | 3 | 4 | 5 |
| 3. The program has political support within the larger organization. | 1 | 2 | 3 | 4 | 5 |
| 4. The program has political support from outside of the organization. | 1 | 2 | 3 | 4 | 5 |
| 5. The program has strong advocacy support. | 1 | 2 | 3 | 4 | 5 |
| **Funding Stability: Establishing a consistent financial base for your program** | **Strongly disagree** | **disagree** | **Neither agree nor disagree** | **agree** | **Strongly agree** |
| 1. The program exists in a supportive state economic setting. | 1 | 2 | 3 | 4 | 5 |
| 2. The program implements policies to help ensure sustained funding beyond current funding cycle | 1 | 2 | 3 | 4 | 5 |
| 3. The program is funded through balanced contribution from variety of sources. | 1 | 2 | 3 | 4 | 5 |
| 4. The program has a combination of stable funding beyond current funding cycle | 1 | 2 | 3 | 4 | 5 |
| 5. The program has flexible, unrestricted funding beyond current funding cycle | 1 | 2 | 3 | 4 | 5 |
| **Partnerships: Cultivating connections between your program and its stakeholders** | **Strongly disagree** | **disagree** | **Neither agree nor disagree** | **agree** | **Strongly agree** |
| 1. Diverse community organizations (government, private, non-profit and community organizations) are invested in the success of the program. | 1 | 2 | 3 | 4 | 5 |
| 2. The program communicates with community leaders from diverse organizations (government, private, non-profit and community organizations) | 1 | 2 | 3 | 4 | 5 |
| 3. Diverse organizations (government, private, non-profit and community organizations) are engaged in the development of program goals | 1 | 2 | 3 | 4 | 5 |
| 4. Local leaders from diverse organizations (government, private, nonprofit and community organizations) are engaged in the development of program activities | 1 | 2 | 3 | 4 | 5 |
| 5. Diverse organizations (government, private, non-profit and community organizations) are engaged in implementation of program activities | 1 | 2 | 3 | 4 | 5 |
| **Organizational Capacity: Having the internal support and resources needed to effectively manage your program and its activities** | **Strongly disagree** | **disagree** | **Neither agree nor disagree** | **agree** | **Strongly agree** |
| 1. The program is well integrated into the operations of the organization. | 1 | 2 | 3 | 4 | 5 |
| 2. Organizational systems are in place to support the various program needs. | 1 | 2 | 3 | 4 | 5 |
| 3. Leadership effectively articulates the vision of the program to external partners. | 1 | 2 | 3 | 4 | 5 |
| 4. Leadership efficiently manages staff and other resources. | 1 | 2 | 3 | 4 | 5 |
| 5. The program has adequate staff to complete the program’s goals. | 1 | 2 | 3 | 4 | 5 |
| **Program Evaluation*:* Assessing your program to inform planning and document results** | **Strongly disagree** | **disagree** | **Neither agree nor disagree** | **agree** | **Strongly agree** |
| 1. The program has the capacity for quality program evaluation. | 1 | 2 | 3 | 4 | 5 |
| 2. The program reports short term, intermediate and long-term outcomes. | 1 | 2 | 3 | 4 | 5 |
| 3. Evaluation results inform program planning and implementation. | 1 | 2 | 3 | 4 | 5 |
| 4. Program evaluation results are used to demonstrate successes to funders and other key stakeholders. | 1 | 2 | 3 | 4 | 5 |
| 5. The program provides strong evidence to the public that the program works. | 1 | 2 | 3 | 4 | 5 |
| **Program Adaptation*:* Taking actions that adapt your program to ensure its ongoing effectiveness** | **Strongly disagree** | **disagree** | **Neither agree nor disagree** | **agree** | **Strongly agree** |
| 1. The program periodically reviews the evidence base. | 1 | 2 | 3 | 4 | 5 |
| 2. The program adapts strategies as needed. | 1 | 2 | 3 | 4 | 5 |
| 3. The program adapts to new science. | 1 | 2 | 3 | 4 | 5 |
| 4. The program proactively adapts to changes in the environment. | 1 | 2 | 3 | 4 | 5 |
| 5. The program makes decisions about which components are ineffective and should not continue. | 1 | 2 | 3 | 4 | 5 |
| **Communications:** Strategic communication with stakeholders and the public about your program | **Strongly disagree** | **disagree** | **Neither agree nor disagree** | **agree** | **Strongly agree** |
| 1. The program has communication strategies to secure and maintain public support from local stakeholders (government, private, non-profit and community organizations). | 1 | 2 | 3 | 4 | 5 |
| 2. Program staff communicate the need for the program to the public. | 1 | 2 | 3 | 4 | 5 |
| 3. The program is marketed in a way that generates interest. | 1 | 2 | 3 | 4 | 5 |
| 4. The program increases community awareness of the issue. | 1 | 2 | 3 | 4 | 5 |
| 5. The program demonstrated its value to  local stakeholders (government, private,  non-profit and community  organizations). | 1 | 2 | 3 | 4 | 5 |
| **Strategic Planning:** Using processes that guide your program’s direction, goals, and strategies | **Strongly disagree** | **disagree** | **Neither agree nor disagree** | **agree** | **Strongly agree** |
| 1. The program plans for future resource needs. | 1 | 2 | 3 | 4 | 5 |
| 2. The program has a long-term financial plan. | 1 | 2 | 3 | 4 | 5 |
| 3. The program has a sustainability plan. | 1 | 2 | 3 | 4 | 5 |
| 4. The program’s goals are understood by all stakeholders (government, private, non-profit and community organizations) | 1 | 2 | 3 | 4 | 5 |
| 5. The program clearly outlines roles and responsibilities for all stakeholders (Government, private, non-profit and community organizations) | 1 | 2 | 3 | 4 | 5 |
