# Supplementary material for: Assessing the sustainability of Rwanda’s mass drug administration program for schistosomiasis and soil-transmitted helminthiasis: A mixed-methods application of the program sustainability assessment tool
Source: PLoS Negl Trop Dis. 2026 Jun 25;20(6):e0014455. doi: 10.1371/journal.pntd.0014455 (PMC13298938; doi:10.1371/journal.pntd.0014455)
Supplement: S2 Appendix — (DOCX) [file pntd.0014455.s002.docx]

**S2 Appendix.** Interview Guide for Capacity for Sustainability Assessment

| Political Support | - Who are your champions or advocates? In what ways do they advocate for the program (or have they advocated, or you hope they will do)? Can you provide an example? - Describe the political support or lack thereof that exists within the organization. Include internal existing policies or recent or impending internal policy changes that support or impede the program’s sustainability. - Describe the political support or lack thereof that exists beyond the organization. Include external existing policies or recent or impending external policy changes that support or impede the program’s sustainability. |
| --- | --- |
| Funding Stability | - Describe the current funding situation. - How will the program be funded after END Fund funding ends? - Describe your efforts to obtain funding, past and future. |
| Partnerships | - What organizations or individuals are invested in the success of the program? Why? - How do you see partner organizations or community members contributing to the sustainability of MDA program? - How important is it to have community leaders involved with or committed to the program? - Are there partnership structures such as coalitions or networks created by the project that may be sustained beyond END Fund funding? If so, describe them. |
| Organizational Capacity | - Describe the internal support and resources you currently have for the MDA program, and how this will change after END-FUND funding ends. - Describe the strengths and weaknesses you see in terms of the organizational capacity to maintain this program. |
| Program Evaluation | - Describe how the program has used evaluation findings to strengthen the program and its sustainability. - Describe how you foresee the role of evaluation after END-FUND funding ends. - Who have you shared the program evaluation results with? |
| Program Adaptation | - Provide an example or two that demonstrates adaptability of the MDA program. - How do you think the MDA program level of adaptability affects its sustainability? |
| Communications | - Describe your communication tools and strategies. - How important is it that the program demonstrates its value to the public? |
| Strategic Planning | - Does the program have a sustainability plan? - If yes, describe the program’s sustainability plan.   - How was it developed?   - What does the plan entail? - Is the plan formalized in writing? - Describe how you foresee MDA program continuing after END-Fund funding. |
